# Briquetting and Remelting of Aspiration Dust Generated During High-Carbon Ferrochrome Crushing in Direct Current Electric Arc Furnaces

**DOI:** 10.3390/ma19061149

**Published:** 2026-03-16

**Authors:** Otegen Sariyev, Maral Almagambetov, Nurzhan Nurgali, Kanat Bilyalov, Bauyrzhan Kelamanov, Dauren Yessengaliyev, Assylbek Abdirashit

**Affiliations:** 1 Department of Metallurgy and Mining, K. Zhubanov Aktobe Regional University, Aktobe 030000, Kazakhstan; 2 ERG Research and Development LLP, Astana 010000, Kazakhstan; maral.almagambetov@erg.kz (M.A.); nurzhan.nurgali@erg.kz (N.N.); kanat.bilyalov@erg.kz (K.B.)

**Keywords:** aspiration dust, high-carbon ferrochrome, briquetting, DC arc furnace, chromium recovery, energy efficiency, waste recycling, circular metallurgy

## Abstract

This study addresses the problem of efficient utilization of aspiration dust (AD) generated during crushing of high-carbon ferrochrome (HCFeCr). To solve this issue, a briquetting technology was proposed, involving aspiration dust blended with dry gas-cleaning dust (20 wt.% as filler) and an organic polymer binder (3 wt.%). The produced briquettes demonstrated high mechanical strength (average 195 kg per briquette in splitting strength and 98% drop resistance), ensuring maximum integrity during transportation and handling. Pilot-industrial remelting of 35 tons of briquettes in a 1.8 MVA direct current electric arc furnace (DC EAF) confirmed the effectiveness of the proposed technology for HCFeCr production. Chromium recovery into the alloy reached 94%, which is 3–4% higher compared to remelting of loose dust. The specific electric energy consumption was 1600 kWh/t, representing a 29% reduction compared to loose dust processing. The produced metal met commercial grades FeCr800–FeCr900 specifications. Additional advantages included elimination of dust formation, reduction in fines generation during crushing of the final metal to 15%, and improved environmental performance. The developed technology represents an economically and environmentally viable solution for comprehensive recycling of ferroalloy dust waste.

## 1. Introduction

The production of high-carbon ferrochrome (HCFeCr) is accompanied by the formation of by-products and secondary metallurgical materials. These include slags, dust from dry and wet gas-cleaning systems, refractory scrap, as well as screenings and fine dust generated during HCFeCr crushing.

Slags from HCFeCr production are fully processed into crushed stone for road construction applications [1] or used as components in refractory products [1,2]. Dust from gas-cleaning systems is agglomerated and returned to ferroalloy furnaces for remelting [3,4]. Refractory scrap is also recycled for the production of shaped refractory products [5] and protective coatings for metallurgical ladles [6]. Screenings from HCFeCr crushing are used for lining casting molds and are partially remelted in specialized units [7].

However, the issue of efficient utilization of aspiration dust (AD) generated during HCFeCr crushing remains unresolved. The annual formation of this dust at two ferroalloy plants in the Republic of Kazakhstan, considering capacity expansion since 2014, amounts to approximately 5000 tons [8,9].

Aspiration dust is highly dispersed, and its remelting in loose form is associated with significant mechanical losses during transportation and furnace charging, oxidation losses, and entrainment into gas-cleaning systems [10]. According to preliminary data, up to 15% of the annual AD volume may be lost to gas-cleaning systems. Therefore, minimizing or eliminating such losses through rational recycling methods is essential.

Common AD recycling methods in metallurgy include agglomeration and remelting [11,12,13,14,15] or processes based on self-propagating high-temperature synthesis (SHS) [16,17]. Certain ferroalloy dusts are used as components in sintering charges [18] or in low-carbon ferrochrome production [19,20]. Study [21] reports the potential production of a complex manganoferrrosilicochrome alloy from aspiration dust generated in HCFeCr and ferrosilicomanganese production.

Nevertheless, these methods have several drawbacks, including technological complexity, multiple processing stages, and strict requirements for the chemical composition of the final product.

Considering the experience of domestic enterprises in processing dry gas-cleaning dust and the limited industrial experience in effective recycling of metallic dusts, this study focuses on the agglomeration of aspiration dust generated during HCFeCr crushing. Briquetting followed by electric arc remelting to produce HCFeCr ingots was selected as the base technology due to its relative simplicity and compatibility with standard metallurgical equipment.

The use of agglomerated dust prevents dust entrainment with off-gases during remelting and reduces irreversible material losses during handling and furnace charging. Therefore, agglomerated materials must exhibit sufficient cold and hot strength [22,23], ensuring integrity throughout all stages of metallurgical processing.

Among agglomeration methods, briquetting was selected as the most suitable technique for materials with low wettability and poor granulation characteristics. Each type of metallurgical raw material has specific technological features in briquette production and binder performance. The selection of binder must consider particle morphology and ensure adequate mechanical strength both at ambient conditions and at metallurgical process temperatures. Furthermore, the binder should not introduce harmful impurities into the alloy, excessively dilute the raw material, or increase slag formation.

Historically, inorganic binders such as cement and bentonite were widely used in ferrous metallurgy. Currently, polymer-based organic binders are gaining popularity and may potentially replace inorganic binders [24]. Polymer binders decompose at high temperatures without releasing hazardous by-products and volatilize completely.

Unlike conventional approaches based on direct remelting and agglomeration using standard binders, this study proposes the use of dry bag-filter dust (DGD) from gas-cleaning systems as a plasticizer and filler during aspiration dust briquetting.

Recent studies have demonstrated the potential of technogenic waste recycling in ferroalloy production through resource-efficient smelting technologies and alternative reduction routes [25,26,27,28].

Pilot-industrial trials of the briquetting technology followed by remelting were conducted to adapt existing briquetting equipment to polymer-based binders, replacing traditionally used sodium silicate, cement, and bentonite. The modified mixture composition enabled the production of briquettes meeting mechanical and thermal strength requirements and confirmed their suitability for metallurgical processing with the production of specification-compliant ferrochrome in direct current electric arc furnaces at pilot-industrial scale.

## 2. Materials and Methods

### Raw Materials Characterization and Industrial Symbiosis Approach

**Figure 1:**
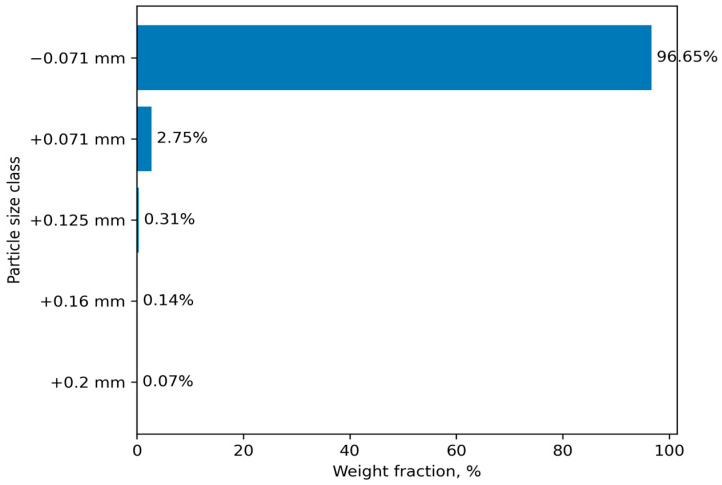
Granulometric composition of aspiration dust.

The primary criteria for evaluating the physicochemical properties of materials include their chemical, phase, and particle size compositions. The data for aspiration dust (AD) according to these criteria are presented below in Table 1 and Figure 1.

The chemical composition of AD was determined using an AXIOSmax Advanced X-ray fluorescence spectrometer (PANalytical, Almelo, The Netherlands). Sulfur and carbon (total carbon and carbonate carbon) were analyzed using a CS-2000 analyzer (Eltra GmbH, Haan, Germany).

The particle size distribution of AD was determined using an ASV-200 (Retsch GmbH, Haan, Germany) sieve analyzer operating under dry sieving conditions.

Based on the content of major elements and impurities, the chemical composition of aspiration dust (AD) is comparable to standard grades of high-carbon ferrochrome (Cr: 65–68 wt.%, Si: 1–2 wt.%, C: 6–9.5 wt.%). Therefore, no significant processing difficulties or deviations are expected, except for silicon, which decreases during secondary electric arc remelting due to its higher susceptibility to oxidation losses.

Particle size analysis indicates that AD is predominantly represented by the fraction below 0.071 mm. In ferrous metallurgy, such fine fractions are typically subjected to granulation followed by high-temperature firing (sintering). However, in the present case, high-temperature sintering is unacceptable due to the high risk of oxidation of the principal element chromium.

Another important factor influencing the selection of processing technology for dispersed materials is particle morphology and structure. Metallographic examination shows that AD particles have irregular, sharp-edged shapes, with the presence of needle-like crystals [30]. Such particle morphology creates difficulties during agglomeration due to the variable contact surface area between the base material and the binder. A low contact area leads to the formation of voids between particles, requiring increased binder consumption to achieve adequate strength [9].

A commonly applied approach in such cases involves the use of filler materials with finer particle size distribution. Fillers help to fill interparticle voids and improve adhesion by increasing the effective contact area between particles and binder. This results in reduced binder consumption and improved mechanical strength of agglomerated materials [29].

Considering that HCFeCr aspiration dust belongs to the above-described type of material (irregular and sharp-edged structure), it is reasonable to use filler materials containing chromium oxides during briquetting in order to minimize dilution of the principal element. From this perspective, dry gas-cleaning dust from ferroalloy furnaces, particularly dust generated during HCFeCr production (DGD), appears promising.

The average chemical composition of this dust, determined by titrimetric analysis, is presented in Table 2.

**Figure 2:**
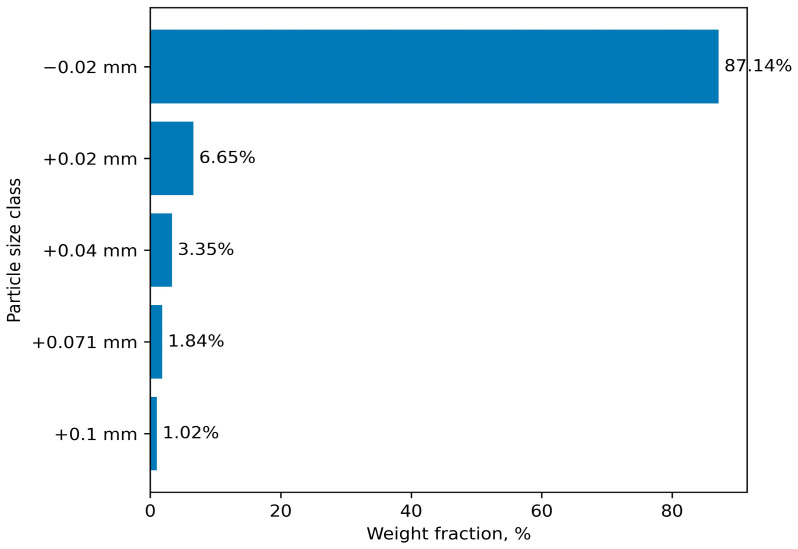
Particle size distribution of dry gas-cleaning dust (DGD).

The particle size distribution of the dust is predominantly represented by the fraction below 0.02 mm, accounting for more than 85% (Figure 2). Particle size analysis was performed using a wet sieving method with a vibratory sieve shaker (Analysette 3 Spartan, Fritsch GmbH, Idar-Oberstein, Germany), as well as a HORIBA LA-950 (HORIBA Ltd., Kyoto, Japan) laser particle size analyzer.

**Figure 3:**
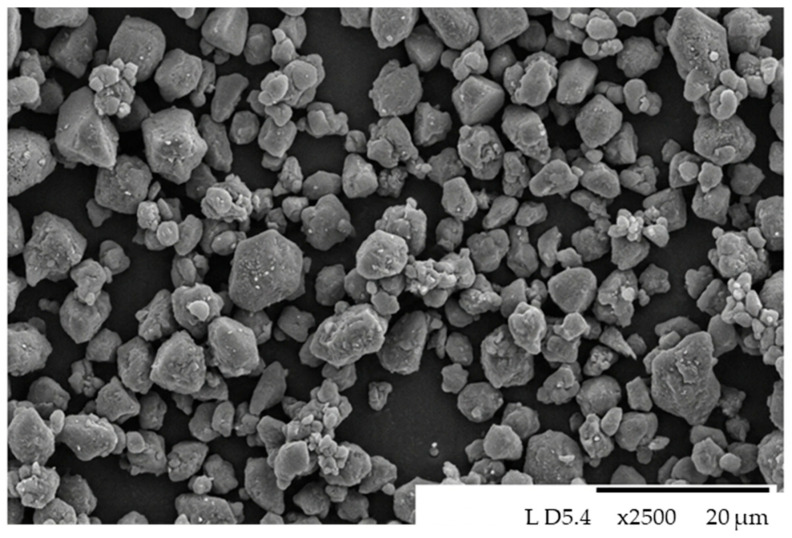
Morphology of dry gas-cleaning dust (DGD) particles under SEM.

Structural analysis of the dry gas-cleaning dust (DGD) sample was carried out using a Hitachi TM-1000 (Hitachi High-Technologies Corporation, Tokyo, Japan) scanning electron microscope. The results showed that the particles exhibit a predominantly spherical morphology (Figure 3). Due to their rounded shape, the particles demonstrate high packing density and effectively fill interparticle voids, resulting in a more compact structure.

**Figure 4:**
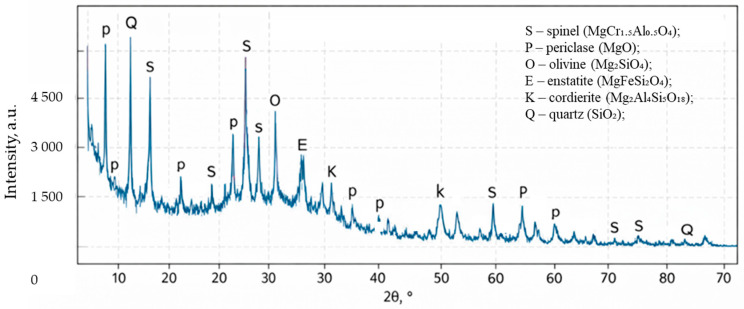
XRD pattern of the dry gas-cleaning dust (DGD) sample with identification of major peaks.

Phase composition analysis of the dust was performed using X-ray diffraction (XRD) (Figure 4) with a DRON-3M digital diffractometer (Bourevestnik JSC, Saint Petersburg, Russia). The results revealed the predominance of spinel, periclase, and olivine phases, along with a minor amount of magnesium aluminosilicates (cordierite).

After obtaining and analyzing all physicochemical characterization data of the raw materials, the study proceeded to the production of a pilot-industrial batch of briquettes.

Preparation of composite briquettes

**Figure 5:**
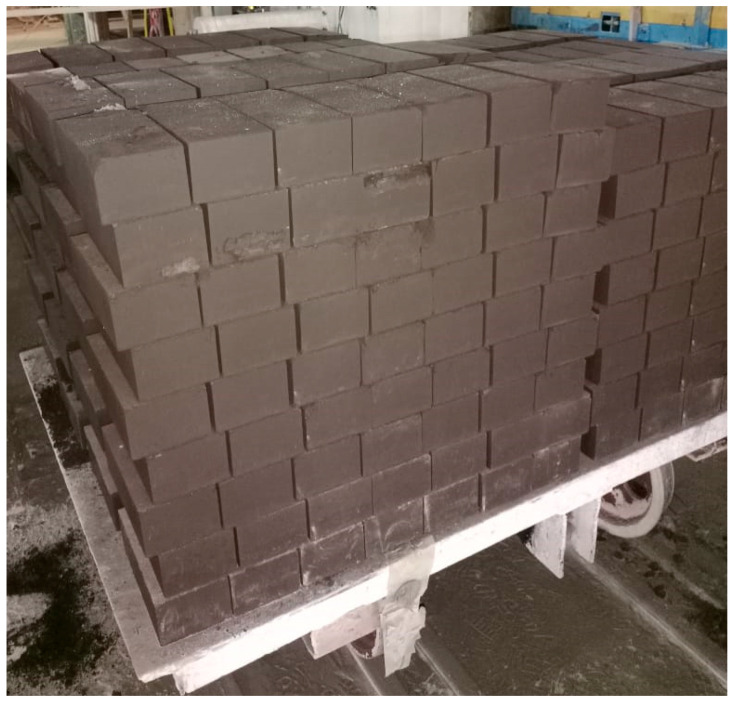
Tray with green briquettes prior to drying.

A pilot-scale batch of briquettes was produced (Figure 5) according to a previously optimized formulation [29,30,31]. The briquetting mixture composition was as follows:

- Aspiration dust (AD): 80 wt.%.
- Dry gas-cleaning dust (DGCD): 20 wt.%.
- Organic polymer binder Ligno 1: 3 wt.% (relative to the dry AD + DGCD mixture).
- Water: 4 wt.% (relative to the total dry mixture).

The briquette preparation process included the following steps:

- Weighing and dry mixing of the components in a twin-screw mixer for 5 min.
- Addition of water followed by wet mixing until complete homogenization (~10 min).
- Briquetting by semi-dry pressing using a hydraulic press (SM 1085A, Stroymash LLC, Chelyabinsk, Russia) to produce briquettes with dimensions of 240 mm × 120 mm × 65 mm.
- Drying of green briquettes in a tunnel furnace at 200 °C for 12 h to ensure complete moisture removal.

According to the above-described methodology, 35 tons of briquettes were produced for subsequent pilot-industrial remelting in a direct current electric arc furnace to obtain high-carbon ferrochrome (HCFeCr). From each produced batch, briquette samples were taken to determine their splitting and drop strength, considering their geometric dimensions.

The average splitting strength of briquettes selected from six batches (10 briquettes per batch) reached 195 kg per briquette, exceeding the required value of 150 kg per briquette. The drop strength (measured as the yield of +5 mm fraction) averaged 98%, compared to the target value of 85%.

In addition to mechanical testing, a microscopic study of the briquette structure was performed using a Zeptools 20 scanning electron microscope (ZEPTools Technology Co., Ltd., Shenzhen, China) to evaluate the influence of the filler material (DGD) and polymer binder on the briquette microstructure. For this purpose, briquette samples were mounted in aluminum holders, embedded in epoxy resin, and polished using diamond abrasive materials.

**Figure 6:**
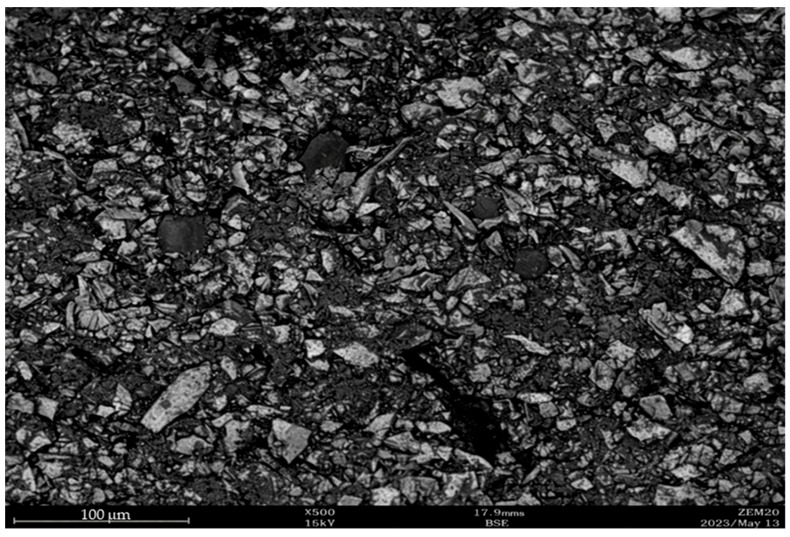
Microstructure of a briquette produced from aspiration dust (AD) and dry gas-cleaning dust (DGD) mixture.

Structural analysis of the samples (Figure 6) showed that the dry gas-cleaning dust acts as a filler-shell due to its high dispersity (87% of the −0.02 mm fraction compared to 96% of the −0.071 mm fraction for AD) and low bulk density (0.2 t/m^3^ versus 2–4 t/m^3^ for AD). These properties allow DGD to almost completely fill the interparticle space between AD particles and to envelop the metallic particles.

The addition of the polymer binder leads to the formation of adhesive films on the surfaces of DGD particles, which in turn bind the AD particles into a rigid framework structure.

This interpretation is consistent with the findings reported in [30], where fine chromite ore was briquetted. The study demonstrated that the polymer binder forms a thin film on the surface of chromite particles, creating an adhesive layer that enhances particle bonding.

Considering that DGD represents dispersed chromite material captured by the gas-cleaning system, it is highly probable that the same strengthening mechanism operates in briquettes produced from the AD–DGD mixture.

The high mechanical strength of the briquettes, including enhanced resistance to splitting and drop impact, prevents dust entrainment and minimizes irreversible material losses during transportation and handling of dispersed AD, as compared to loose remelting. This contributes to more complete incorporation of AD into the remelting process with minimal losses.

## 3. Results

### Remelting of Briquettes Produced from Aspiration Dust

Pilot-industrial briquetting tests of aspiration dust generated during high-carbon ferrochrome (HCFeCr) crushing demonstrated that the produced briquettes possess sufficient mechanical strength for transportation, storage, and charging into metallurgical units. The next stage of the study was to evaluate the feasibility of their pilot-industrial remelting in direct current electric arc furnaces with maximum chromium recovery.

Remelting of aspiration dust in loose form had previously been investigated under pilot-industrial conditions [10]. It was found that charging loose dust into the furnace resulted in significant material losses due to dust entrainment and chromium balance deviations. With chromium recovery levels of approximately 89–90%, a high balance discrepancy (up to 8%) and increased metal losses to slag were observed. Moreover, charging loose dust caused excessive dusting in the working area and required additional gas-cleaning capacity.

The use of briquettes is expected to eliminate these drawbacks. Agglomerated material has a reduced surface area exposed to chromium oxidation and minimal susceptibility to entrainment of fine particles. As a result, improved chromium recovery into the alloy, reduced specific energy consumption, and lower losses to slag and dust can be anticipated.

To evaluate the effectiveness of the proposed technology, pilot-industrial remelting trials of briquettes were conducted in a 1.8 MVA direct current electric arc furnace (DC EAF) for HCFeCr production.

For the pilot trials, briquettes were produced from a mixture of aspiration dust (80 wt.%) and dry gas-cleaning dust (20 wt.%) with the addition of 3 wt.% organic polymer binder. The briquettes measured 240 mm × 120 mm × 65 mm and exhibited an average splitting strength of 195 kg per briquette, ensuring structural integrity during transportation and furnace charging.

**Figure 7:**
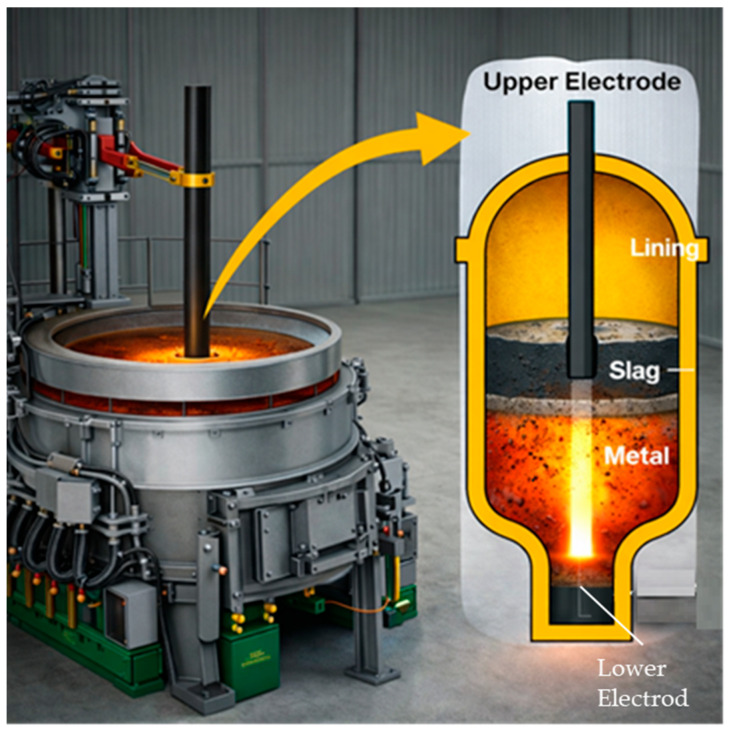
Direct current electric arc furnace DPPT-1.8 MVA.

Remelting was performed in a 1.8 MVA direct current electric arc furnace with a bath capacity of approximately 1 ton. The furnace was a closed-type unit equipped with one upper graphite electrode and one bottom electrode (Figure 7). The working lining was composed of magnesia refractory material. Metal and slag tapping were carried out simultaneously by tilting the furnace toward the tap hole.

The charge was introduced in 1-ton batches. The composition of one heat included:

- Briquettes produced from aspiration dust: 700–800 kg;
- Return slag from HCFeCr production: 150–200 kg;
- Reducing agent (coke fines): 20–30 kg.

The furnace was initially preheated by charging a small amount of coke under the electrode. After reaching the operating regime, sequential charging of briquettes and slag was performed.

Due to their high mechanical strength, the briquettes maintained their shape within the charge column and did not disintegrate during feeding, which significantly reduced dust formation and entrainment losses.

## 4. Discussion

Pilot-industrial remelting trials of briquettes produced from aspiration dust were conducted in a 1.8 MVA direct current electric arc furnace (DC EAF). During the campaign, 35 tons of briquettes were processed, resulting in the production of commercial high-carbon ferrochrome grades FeCr800–FeCr900.

The trials demonstrated that the use of briquettes ensured a more stable thermal regime and reduced specific electricity consumption. The average value was 1600 kWh/t (Table 3), which is 29% lower than that observed during remelting of loose dust.

Raw material consumption and metal yield for selected heats are presented in Table 4. The average furnace productivity was approximately 10 t of metal per day.

The summarized technical indicators are presented in Table 5. A high chromium recovery of 94% was achieved, which is 4–5% higher than in the case of loose dust remelting. The improvement is attributed to reduced dust formation during charging and lower chromium losses to slag.

The chemical composition of the produced metal is given in Table 6. The alloy complied with FeCr800–FeCr900 grade requirements: Cr content was approximately 71%, C about 8%, and Si did not exceed 0.6%. Harmful impurities were maintained at low levels (S ≤ 0.03%, P ≤ 0.02%).

The chromium balance (Table 7) confirmed that the major portion of chromium was concentrated in the metal, while slag losses were minimized, demonstrating the high efficiency of briquette application compared to loose dust processing.

After crushing and screening of the ingots at the Aktobe Ferroalloy Plant finishing shop, the yield of fines (<10 mm fraction) was approximately 15% (Table 8), which is significantly lower than the typical 20–25% for commercial ferrochrome.

The reduced fines formation is likely attributed to the increased density of the metal after remelting, as well as the fact that during casting the metal remains under a protective slag layer, reducing thermal stresses and brittleness. Thus, the use of briquetted raw materials improves not only metallurgical performance but also the physical properties of the final product.

The pilot-industrial trials demonstrate the high efficiency of remelting briquettes produced from aspiration dust in direct current furnaces. The technology enables significant improvement in chromium recovery (up to 94%) and reduction in specific energy consumption to 1600 kWh/t. Additional advantages include reduced dust formation and associated losses, improved environmental performance, and lower generation of fines during subsequent crushing.

Collectively, these results confirm the high economic and environmental feasibility of implementing the proposed technology.

Environmental assessment of ferroalloy waste streams and slag processing routes confirms the importance of integrated recycling approaches [32].

## 5. Conclusions

This study addressed the problem of efficient recycling of aspiration dust generated during crushing of high-carbon ferrochrome (HCFeCr). A briquetting method was proposed using dry gas-cleaning dust as an additive and an organic polymer binder. The produced briquettes exhibited high mechanical strength (average 195 kg per briquette), maintained their structural integrity during transportation and charging, and demonstrated suitability for remelting in a direct current electric arc furnace.

Pilot-industrial remelting trials in a 1.8 MVA DC electric arc furnace confirmed the technological and economic feasibility of incorporating aspiration dust into HCFeCr production. During the trial campaign, 35 tons of briquettes were produced and remelted. The following results were achieved:

- Chromium recovery reached 94%, with 30 tons of ferrochrome produced;
- Specific electricity consumption was 1600 kWh/t, representing a 29% reduction compared to loose dust remelting;
- The produced alloy met FeCr800–FeCr900 grade specifications (Cr ~71%, C ~8%, low S and P content);
- Fines generation during crushing was limited to 15%, which is lower than typical values (20–25%).

The use of briquettes minimized dust formation during charging and reduced chromium losses to slag, ensuring a more stable smelting process.

Thus, the developed technology of aspiration dust briquetting followed by remelting into high-carbon ferrochrome ingots represents an effective solution for ferroalloy waste recycling. The process provides high chromium recovery, reduced energy consumption, improved product quality, and decreased environmental impact. The technology demonstrates strong industrial potential for implementation at operating ferroalloy plants.

## Figures and Tables

**Table 1:** Chemical composition of aspiration dust (wt.%) [29].

Cr	Si	C	S	P	Fe
64.89	2.54	8.24	0.027	0.014	24.289

**Table 2:** Chemical composition of dry bag-filter dust (DGD), wt.% [29].

MgO	SiO_2_	CaO	Cr_2_O_3_	Al_2_O_3_	Fe_total_	C
32.8	18.0	0.4	14.9	6.8	4.2	5.8

**Table 3:** Specific electricity consumption for ferrochrome smelting.

Heat No.	Furnace Operating Time, h	Metal Produced, kg	Specific Electricity Consumption, kWh/
1	2.0	820	1580
10	1.9	810	1640
25	2.1	840	1635
33	2.05	845	1560
43	2.0	825	1545
Average	2	~828	1600

**Table 4:** Raw material consumption and metal yield.

Heat No.	Briquettes, kg	Slag, kg	Coke, kg	Metal Produced, kg	Slag Produced, kg
1	780	160	25	820	200
10	790	170	22	810	210
25	800	180	28	840	220
33	805	175	26	845	215
43	785	165	24	825	205
Average	~792	~170	~25	~828	~210

**Table 5:** Technical performance indicators of ferrochrome smelting in the 1.8 MVA DC EAF.

Parameter	Value
Total briquettes charged, kg	35,000
Total slag charged, kg	7500
Total coke charged, kg	1000
Metal produced, kg	30,000
Slag produced, kg	8500
Chromium recovery, %	94.0
Specific electricity consumption, kWh/t	1600
Average tap weight, kg	~828
Daily productivity, t	about 10

**Table 6:** Detailed chemical composition of smelting products (wt.%).

Sample	Metal						Slag				
	Cr	C	Si	Fe	S	P	Cr_2_O_3_	MgO	Al_2_O_3_	SiO_2_	FeO
1	70.3	8.39	0.65	8.39	0.038	0.017	6.2	32.9	16.7	33.4	1.6
2	71.6	8.11	0.68	8.11	0.033	0.02	7.7	33.5	16.7	33.0	1.4
3	71.3	8.38	0.46	8.38	0.03	0.018	6.5	29.3	15.9	30.8	0.6
Average	71.016	8.12	0.52	8.14	0.03	0.01	6.7	32.0	16.3	32.5	1.09

**Table 7:** Chromium balance, kg.

Chromium Source	Mass, kg	Chromium Distribution	Mass, kg
From briquettes	22,712	In metal	21,340
From slag	2839	In slag	2933
Total input	25,551	Total output	24,273
Balance discrepancy			1278 kg (5.0%)
Chromium recovery Cr, %			94.0

**Table 8:** Screening results of pilot-produced metal.

Fraction, mm	Mass, kg	Share, %
0–10	4350	15.0
10–50	21,170	73.0
50–150	4480	12.0
Total	30,000	100%

## Data Availability

The raw data supporting the conclusions of this article will be made available by the authors on request.
